# Acute pancreatitis in intraductal papillary mucinous neoplasm: a single-center retrospective cohort study with systematic review and meta-analysis

**DOI:** 10.1186/s12876-023-02972-4

**Published:** 2023-12-01

**Authors:** Ji-Hao Xu, Chu-Yan Ni, Yan-Yan Zhuang, Li Li, Ying Lin, Zhong-Sheng Xia, Wei-Rong Wu, Qi-Kui Chen, Wa Zhong

**Affiliations:** 1 Department of Gastroenterology, Sun Yat-Sen Memorial Hospital, Sun Yat-Sen University, 107 Yan Jiang Xi Road, Guangzhou, 510120 Guangdong People’s Republic of China; 2 Department of Gastroenterology, Longgang District People’s Hospital of Shenzhen, Central City of Longgang District, No.53 Aixin Road, Shenzhen City, 518172 Guangdong People’s Republic of China; 3 Department of Emergency, Sun Yat-Sen Memorial Hospital, Sun Yat-Sen University, 107 Yan Jiang Xi Road, Guangzhou, 510120 Guangdong People’s Republic of China

**Keywords:** Acute pancreatitis (AP), Malignant intraductal papillary mucinous neoplasm (IPMN), High grade dysplasia (HGD)

## Abstract

**Background:**

Intraductal papillary mucinous neoplasm (IPMN) is a cystic tumor of the pancreas arising from abnormal papillary proliferation of ductal epithelial cells, and is a precancerous lesion of pancreatic malignancy. This study aimed to evaluate associations between acute pancreatitis (AP) and histologic subtypes of IPMN.

**Methods:**

In the clinical study, patients with IPMN confirmed by surgical resection specimens at our institute between 2009 and 2021 were eligible for inclusion. Associations and predictive accuracy of AP on the presence of HGD were determined by logistic regressions. In addition, a systematic review and meta-analysis was conducted through literatures upon search in PubMed, Embase, CENTRAL, China National Knowledge Infrastructure (CKNI), and Wanfang database, up to June, 2023. Pooled effects of the associations between AP and HGD and intestinal epithelial subtype subtype, shown as odds ratios (ORs) with 95% confidence intervals (CIs), were calculated using random effects model.

**Results:**

The retrospective cohort study included 47 patients (32 males, 15 females) diagnosed with IPMN at our center between 2009 and 2021, including 11 cases with AP (median 62 years) and 36 cases (median 64.5 years) without. Accuracy, sensitivity, specificity, positive predictive value (PPV), and negative predictive value (NPV) of AP in predicting HGD were 78.7%, 57.1%, 82.5%, 36.4%, and 91.7%, respectively. Univariate logistic regression analysis showed that AP group had greater odds of presence of HGD (OR: 6.29,95% CI: 1.14–34.57) than non-AP group. Meta-analysis of five case-control studies in the literature included 930 patients and showed that AP-IPMN patients had higher odds for HGD (OR: 2.13, 95% CI 1.38–3.29) and intestinal epithelial subtype (OR: 5.38, 95% CI: 3.50–8.27) compared to non-AP IPMN.

**Conclusions:**

AP is predictive of malignancy in patients with IPMN.

**Supplementary Information:**

The online version contains supplementary material available at 10.1186/s12876-023-02972-4.

## Background

IPMN is a cystic tumor of the pancreas characterized by abnormal papillary growth of ductal epithelial cells and mucus production [1]. It is considered a precursor to pancreatic malignancy. It is often asymptomatic, although some cases may present with symptoms such as abdominal pain, weight loss, steatorrhea, new-onset diabetes, pancreatitis, or obstructive jaundice [1].

The incidence and diagnosis of IPMN have been increasing over the past three decades, particularly among older adults, partly due to advancements in imaging technology [2]. IPMN can be classified into different types based on the location of the lesion, namely main duct, branch duct, and mixed type [3]. According to the 2010 WHO classification criteria, IPMN can be further categorized into low-grade dysplasia (LGD), intermediate-grade dysplasia (MGD), high-grade dysplasia (HGD), and invasive carcinoma (IC) [4]. Furthermore, based on the histologic characteristics of tumor epithelial cells, IPMN can be divided into four subtypes: gastric, intestinal, pancreaticobiliary, and eosinophilic [5–9].

Acute pancreatitis is an inflammatory response of the pancreatic tissue caused by the activation of pancreatic enzymes, and it has been observed that AP is closely associated with pancreatic tumors [10]. A proportion of unexplained AP cases, around 9%, are caused by tumors, and some tumors present with AP as their initial manifestation [11, 12]. Studies have shown varying associations between AP and IPMN histology. For instance, Venkatesh et al. reported that 12–67% of IPMN patients experience AP, and 7–34.6% of AP cases are associated with IPMN [13]. However, the relationship between AP and specific histologic subtypes of IPMN remains uncertain. Jang et al. found that AP due to IPMN is more frequent in the competent and mixed subtypes, while Hata et al. reported that most AP cases associated with IPMN are from the intestinal subtype [14, 15]. Other studies have explored the association between AP and the degree of histologic heterogeneity in IPMN, yielding conflicting results [16–21].

There is a lack of comprehensive studies on the clinical characteristics of IPMN patients in China. Therefore, our study aimed to fill this gap by conducting a cohort study to investigate the associations between AP and malignant IPMN histology in a single center in southern China, with a systematic review and meta-analysis. By clarifying the relationship between AP and specific histologic subtypes of IPMN, we hope to provide insights for improving these conditions’ understanding, diagnosis, and management.

## Methods

### Clinical study

#### Study design and sample

Patients with IPMN were confirmed by surgical resection specimens at our hospital between January 2009 and January 2021. Patients with AP induced by gallstones, hyperlipidemia, autoimmunity, pancreas malformation, iatrogenesis, or alcohol abuse were excluded. All data, including demographic, clinical, and histological characteristics, were collected from medical records.

Indications for surgery were: IPMN diagnosed by image (CT or MRI) and met one or more of the following: (1) main pancreatic duct (MPD) dilation ≥ 10 mm; (2) combined with obstructive jaundice; (3) enhanced wall nodules ≥ 5 mm; 4). complicated with acute or chronic pancreatitis; 5) cachexia without known causes; and 6) upper abdominal pain that cannot be relieved by active medical treatment and without other known causes.

All IPMN diagnoses were histologically confirmed as cystic or solid lesions or pancreatic duct dilatation. On the other hand, the diagnosis of AP, according to the revised Atlanta Classification, [22] requires two of the following findings: (1) typical abdominal pain of AP; (2) serum lipase activity (and/or amylase activity) at least three times higher than the upper limit of normal value; (3) characteristics of AP on computer tomography (CT), magnetic resonance imaging (MRI), or sonographic findings.

The outcomes of interest were the predictive performance of AP on HGD, as well as the association between AP-IPMN (versus non-AP-IPMN) and the presence of HGD.

#### Ethics considerations

The study protocol was approved by the Institutional Review Board of Sun Yat-Sen Memorial Hospital (approval number: SYSEC-KY-KS-2021-017), and informed consent of included patients was waived due to the study’s retrospective nature.

### Systematic review and Meta-analysis

#### Search strategy and selection criteria

This systematic review and meta-analysis were conducted in accordance with the Preferred Reporting Items for Systematic Reviews and Meta-Analyses (PRISMA) guidelines [23]. PubMed, Embase, CENTRAL, CKNI, and Wanfang databases were searched, up to June 1, 2023. The search terms and the combinations were: (“IPMN” OR “intraductal papillary mucinous neoplasm” OR “IPMT” OR “intraductal papillary mucinous tumor” OR “IPMA” OR “intraductal papillary mucinous adenoma” OR “IPMC” OR “intraductal papillary mucinous carcinoma”) AND (“pancreatitis” OR “acute pancreatitis” OR “AP”). No filters were applied in the search.

#### Inclusion and exclusion criteria

The inclusion criteira of this systematic review and meta-analysis was performed in accordance with the PECO criteria (participants, exposure, comparison, and outcomes). P: patients with IPMN confirmed by histopathology or image, E: AP group; C: non-AP group; O: presence of HGD or intestinal epithelial subtype. There was no language restriction for the study eligibility.

Exclusion criteria were: reviews / case reports / meeting abstracts; non-human studies, studies without outcomes of interest; patients not meeting IPMN or AP diagnostic criteria; and patients’ AP was due to other causes such as gallstones, alcohol, high triglycerides, autoimmune diseases, metabolic diseases, or pancreas anatomical abnormalities.

#### Data extraction

Two investigators independently searched the literature, screened and evaluated the included studies, and extracted relevant data. In disagreements, the third author and the two investigators discussed and agreed through consultation. Extraction of the study data included authors, year of publication, study site, study type, sample size, age, sex, presence of pancreatitis (number of episodes, severity, etiology), IPMN characteristics (location, typing, degree of histiocytic heterogeneity, epithelial cell subtype), treatment method, follow-up, and prognosis.

#### Quality Assessment

The Newcastle-Ottawa Scale (NOS) was used to assess the quality of the studies included [24]. The NOS scale assigns a maximum of nine points to each study, with four points awarded for acceptable participant selection, two points awarded for participant comparability based on design and analysis, and three points awarded for adequate outcome ascertainment. Studies with five or more points were considered high quality. Two independent reviewers conducted the quality assessment. A third reviewer was consulted if uncertainties existed.

### Statistical analysis

For clinical data analysis, categorical data are presented as n (%) and analyzed using Fisher’s exact test. Continuous data are presented as the median (Q1-Q3) and analyzed by the Wilcoxon rank sum test. Univariate and multivariate logistic regression models were used to estimate odds ratios (ORs) and 95% confidence intervals (CIs). All statistical tests were two-sided with a significance level of 0.05. Data management and statistical analyses were conducted using SAS version 9.4 software (SAS Institute, Inc.).

The χ2 test for homogeneity was performed for meta-analysis, and heterogeneity between studies was assessed using Cochran Q and I^2^ statistics. A random-effects model was used if the I^2^ statistic was more than 50%. When a few studies were included in the meta-analysis, the statistical power of the heterogeneity test was low [25] and a random effects model was used [26]. Pooled effects were calculated and shown as ORs and 95% CIs, with bilateral p-values < 0.05 considered statistically significant. The meta-analysis used Review Manager (version 5.3, Cochrane Collaboration).

## Results

### Clinical study

A total of 47 patients with IPMN were included in this study, 11 with AP-IPMN and 36 with non-AP IPMN. In the non-AP group, 25 patients were symptomatic, in which 8 had obstructive jaundice, 1 had calchexia, and 16 had abdominal pain not relieved by active medical treatment.

Baseline characteristics are shown in Table 1. The median age was 62.0 (Q1-Q3: 52.0–72.0) for the AP-IPMN group and 64.5 (Q1-Q3: 57.5–69.5) for the non-AP IPMN group. No significant differences were found in family history and laboratory values between the two groups. However, smoking (p = 0.033) and hypertension (p = 0.039) were more prevalent in the non-AP than AP group.

**Table 1:** Baseline demographic and clinical characteristics in patients with IPMN with/without AP

	IPMN		P-value
	AP (n = 11)	Non-AP (n = 36)	
**Demography**			
Age	62.0 (52.0–72.0)	64.5 (57.5–69.5)	0.555
Sex			0.461
Male	9 (81.8)	23 (63.9)	
Female	2 (18.2)	13 (36.1)	
Smoking	1 (9.1)	17 (47.2)	**0.033**
Alcohol consumption	0 (0)	4 (11.1)	0.560
Hypertension	1 (9.1)	16 (44.4)	**0.039**
Diabetes	1 (9.1)	8 (22.2)	0.663
**Family History**			
AP	0 (0)	0 (0)	
Pancreatic tumors	0 (0)	0 (0)	
**AP occurrence times**			
1	8 (72.7)		
2	1 (9.1)		
3	2 (18.2)		
**Laboratory results**			
Total bilirubin (µmol/L)	11.8 (8.2–14.8)	14.3 (10.3–42.2)	0.158
CEA (ng/ml)	1.5 (1.2–3.2)	2.5 (1.5–4.4)	0.299
CA199 (ng/ml)	15.3 (7.3–29.5)	12.9 (8.9–44.7)	0.915

AP: acute pancreatitis; CA: carbohydrate antigen; CEA: carcinoembryonic antigen; IPMN: intraductal papillary mucinous neoplasms

Significant values are shown in bold.Continuous data are presented as median (Q1-Q3) and categorical data are presented as n (%)

The clinical characteristics of patients in the AP-IPMN and non-AP-IPMN groups are shown in Table 2. Radiology findings revealed no significant differences between the two groups in terms of tumor site, tumor type, main pancreatic duct (MPD) size, cyst/mass size, presence of reinforced wall nodules, reinforced cyst wall, and calcification (all P > 0.05). In addition, no significant differences were found between the two groups regarding pathologic features in the epithelial subtype, degree of dysplasia, chronic pancreatitis, and glandular atrophy (all P > 0.05).

**Table 2:** Characteristics of radiologic imaging and pathology in patients with IPMN with/without AP

	IPMN		P-value
	AP (n = 11)	Non-AP (n = 36)	
**Radiologic imaging**			
IPMN Site			1.000
Body and tail	2 (18.2)	6 (16.7)	
Head and neck	9 (81.8)	30 (83.3)	
IPMN type			0.785
MD	1 (9.1)	4 (11.1)	
MIX	3 (27.3)	14 (38.9)	
BD	7 (63.6)	18 (50.0)	
MPD			0.435
< 10 mm	7 (63.6)	28 (77.8)	
≥ 10 mm	4 (36.4)	8 (22.2)	
Cystic			
< 30 mm	7 (63.6)	13 (38.2)	0.176
≥ 30 mm	4 (36.4)	21 (61.8)	
missing	0	2	
Tuberous enhancement	3 (27.3)	8 (22.2)	0.703
Cystic wall enhancement	3 (27.3)	13 (36.1)	0.725
Calcification	3 (27.3)	4 (11.1)	0.330
**Pathology**			
Epithelial subtype			0.494
Gastric	0 (0)	6 (21.4)	
Intestinal	3 (42.9)	6 (21.4)	
Pancreatobiliary	3 (42.9)	13 (46.4)	
Oncocytic	1 (14.3)	3 (10.7)	
Unknown	4	8	
Degree of dysplasia			0.213
LGD	2 (18.2)	11 (30.6)	
MGD	3 (27.3)	14 (38.9)	
HGD	4 (36.4)	3 (8.3)	
IC	2 (18.2)	8 (22.2)	
Chronic pancreatitis	3 (27.3)	14 (38.9)	0.722
Glandular atrophy	2 (18.2)	8 (22.2)	1.000

AP: acute pancreatitis; BD: branch duct subtype; HGD: high grade dysplasia; IC: invasive cancer; IPMN: intraductal papillary mucinous neoplasms; LGD: low grade dysplasia; MD: main duct subtype; MIX: mixed type; MGD: moderate grade dysplasia; MPD: main pancreatic duct

Continuous data are presented as median (Q1-Q3) and categorical data are presented as n (%)

Tools for sensitivity, specificity, PPV, NPV, and accuracy were analyzed using MPD size, jaundice, tuberous enhancement, and AP as predictors of the degree of dysplasia and intestinal epithelial subtype (Table 3). Among patients with IPMN, patients with AP had the highest PPV (36.4%), NPV (91.7%), and accuracy (78.7%) for HGD when compared to the results of the other three tools. In addition, the sensitivity of AP in predicting HGD (57.1%) was the same as that of MPD ≥ 10 mm. The specificity of AP in predicting HGD (82.5%) was the same as the specificity for jaundice. However, AP was less predictive for invasive cancer, malignant (HGD + IC), and intestinal epithelial subtypes than MPD ≥ 10 mm or jaundice.

**Table 3:** Sensitivity, specificity, PPV, NPV, and accuracy in predicting degree of dysplasia in patients with IPMN

	Sensitivity	Specificity	PPV	NPV	Accuracy
HGD					
MPD ≥ 10 mm	57.1%	80.0%	33.3%	91.4%	76.6%
Jaundice	14.3%	82.5%	12.5%	84.6%	72.3%
Tuberous enhancement	42.9%	80.0%	27.3%	88.9%	74.5%
AP	57.1%	82.5%	36.4%	91.7%	78.7%
IC					
MPD ≥ 10 mm	40.0%	78.4%	33.3%	82.9%	70.2%
Jaundice	50.0%	91.9%	62.5%	87.2%	83.0%
Tuberous enhancement	30.0%	78.4%	27.3%	80.6%	68.1%
AP	20.0%	75.7%	18.2%	77.8%	63.8%
Malignant (HGD + IC)					
MPD ≥ 10 mm	47.1%	86.7%	66.7%	74.3%	72.3%
Jaundice	35.3%	93.3%	75.0%	71.8%	72.3%
Tuberous enhancement	35.3%	83.3%	54.5%	69.4%	66.0%
AP	35.3%	83.3%	54.5%	69.4%	66.0%
Intestinal epithelial subtype					
MPD ≥ 10 mm	55.6%	81.6%	41.7%	88.6%	76.6%
Jaundice	33.3%	86.8%	37.5%	84.6%	76.6%
Tuberous enhancement	33.3%	78.9%	27.3%	83.3%	70.2%
AP	33.3%	78.9%	27.3%	83.3%	70.2%

AP: acute pancreatitis; HGD: high grade dysplasia; IC: invasive cancer; IPMN: intraductal papillary mucinous neoplasms; MPD: main pancreatic duct; NPV: negative predictive value; PPV: positive predictive value

Association AP and the degree of dysplasia and intestinal epithelial subtype, eavluated by surgical specimens, were further analyzed (Table 4). The proportion of developing HGD was significantly higher in the AP-IPMN group (36.4%) than in the non-AP IPMN group (8.3%) (p = 0.042). Univariate logistic regression analysis showed that the risk of developing HGD was significantly higher in the AP-IPMN group than in the non-AP IPMN group (OR: 6.29; 95% CI: 1.14–34.57). However, multivariable logistic regression analysis revealed no significant associations between AP and HGD.

**Table 4:** AP effects on degree of dysplasia and intestinal epithelial subtype in patients with IPMN

Outcomes	IPMN		P-value	Univariate			Multivariate	
	AP (n = 11)	Non-AP (n = 36)		OR	95% CI		aOR	95% CI
HGD	4 (36.4)	3 (8.3)	**0.042**	**6.29**	**1.14–34.57**		3.92	0.62–24.85
IC	2 (18.2)	8 (22.2)	1.000	0.78	0.14–4.35		0.69	0.11–4.44
Malignant (HGD + IC)	6 (54.6)	11 (30.6)	0.171	2.73	0.68–10.87		2.00	0.44–9.07
Intestinal epithelial subtype	3 (27.3)	6 (16.7)	0.419	1.88	0.38–9.20		1.92	0.31–11.83

AP: acute pancreatitis; CI: confidence interval; HGD: high grade dysplasia; IC: invasive cancer; IPMN: intraductal papillary mucinous neoplasm; aOR: adjusted odds ratio

Adjusted for smoking and hypertension

Significant values are shown in bold

Perioperative outcomes in patients having IPMN with and without AP are documented in Supplementary Table 1. The median postoperative hospitalization days in the AP-IPMN group was 8.0 (Q1-Q3: 6.0–19.0), which was significantly less than the median postoperative hospitalization days in the non-AP IPMN group of 21.0 (Q1-Q3: 11.0–39.0) (p = 0.014). No significant differences were found in postoperative complications between the two groups. However, the proportion of tumor recurrence after hospital discharge was significantly higher in the AP-IPMN group (27.3%) than in the non-AP IPMN group (2.9%) (p = 0.037).

### Meta-analysis

**Fig. 1:**
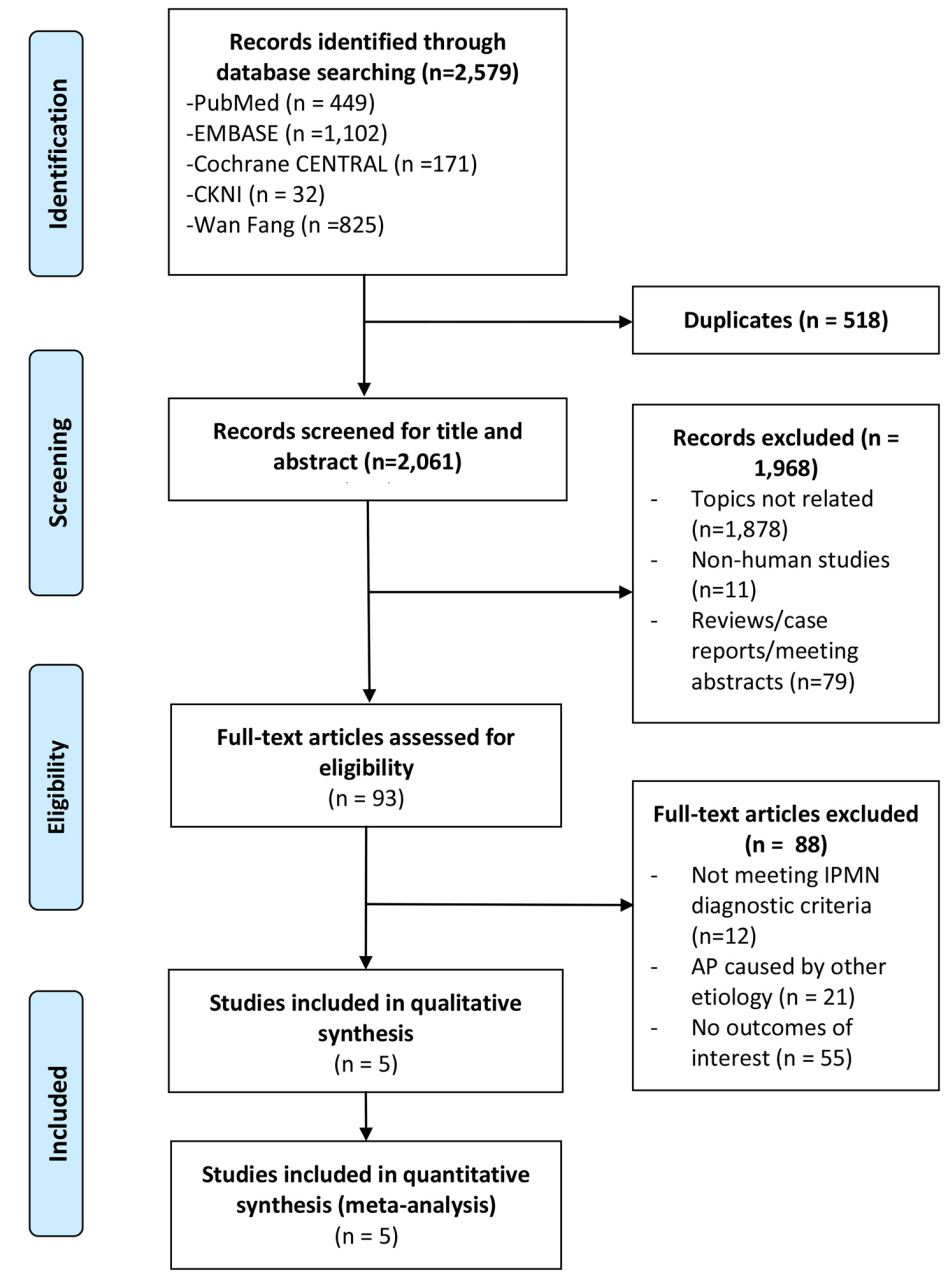
Flow chart of literature search and study inclusion

The PRISMA flow diagram of study selection process is shown in Fig. 1. Initially 2,579 records were retrieved through search, after exclusion, five articles were included in the qualitative and quantitative synthesis (Fig. 1).

The five studies included were all case-control designs, with a total of 930 patients who had histologically confirmed IPMN. Among which, 184 (19.8%) had AP and 746 (80.2%) had not. Characteristics of the two groups are shown in Table 5. The mean age at diagnosis was 66.1 years, and 54.5% were males. The follow-up duration ranged from 1 to 168 months. The NOS scale scores of the included studies, as summarized in Table 5, were 6 to 8, upon which all studies were considered high quality. Details of study quality in each domain of NOS are documented in Supplementary Table 2.

**Table 5:** Characteristics of the selected studies

**Study**	**Country**	**Study type**	**Groups**	**Age** ^**a**^	**Gender (M/F)**	**Follow-up (months)** ^**a**^	**Diagnosis of AP**	**Diagnosis of IPMN**	**NOS score**
Tanaka 2020	Japan	Case control study	AP: 20	69 (43 ~ 81)	12/8	48 (12 ~ 144)	Japanese guidelines; strict control of confounding factors	Histology; Consensus Guideline	8
			non-AP: 162	69 (43 ~ 86)	96/66				
Morales-Oyarvide 2015	USA	Case control study	AP: 69	61 ± 12.4	35/34	58 (2 ~ 108)	Medical records; basic control of confounding factors	Histology; Consensus Guideline	6
			non-AP: 256	70 ± 9.5	124/132				
Hata 2013	Japan	Case control study	AP: 12	65 (35 ~ 73)	7/5	Not available	The Atlanta Standard; strict control of confounding factors	Histology; Consensus Guideline	7
			non-AP: 76	65.5(41 ~ 82)	47/29				
Tsutsumi 2010	Japan	Case control study	AP: 19	70.0 ± 8.2	11/8	Not available	JPN standard; basic control of confounding factors	Histology; Consensus Guideline	7
			non-AP: 131	66.0 ± 8.6	82/49				
Pelletier 2010	France	Case control study	AP: 64	65 (38 ~ 75)	49/15	24 (1 ~ 168)	French Consensus; Strict Control of Confounding Factors	Histology	6
			non-AP: 121	57 (28 ~ 75)	44/77				

AP: acute pancreatitis; IPMN: intraductal papillary mucinous neoplasms; NOS, Newcastle-Ottawa Scale

^a^ Median (range) or mean ± SD

**Fig. 2:**
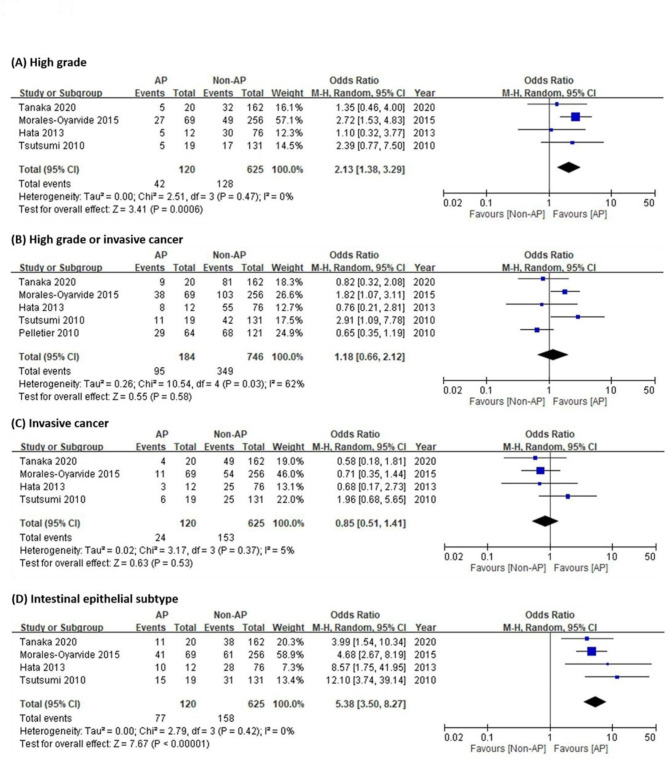
Forest plots for degree of dysplasia and intestinal epithelial subtype in intraductal papillary mucinous neoplasms (IPMN)

Figure 2 shows the meta-analysis of the two groups of patients with AP-IPMN and non-AP IPMN, evaluating the different degrees of dysplasia and development of the intestinal epithelial subtype in IPMN. The random effects model was applied for various degrees of dysplasia and development of intestinal epithelial subtype (high grade: I^2^ = 0%, P < 0.001; high grade or invasive cancer: I^2^ = 62%, P = 0.58; invasive cancer: I^2^ = 5%, P = 0.53; intestinal epithelial subtype: I^2^ = 0%, P < 0.001). The pooled analysis showed that patients with AP-IPMN had an increased risk of high grade compared to non-AP IPMN (OR: 2.13; 95% CI: 1.38–3.29). In addition, patients with AP-IPMN had a higher risk of developing an intestinal epithelial subtype than non-AP IPMN patients (OR: 5.38; 95% CI: 3.50–8.27).

## Discussion

The present study investigated IPMN, the most common cystic tumor of the pancreas, [24] in patients with AP, finding that patients with AP IPMN had a higher risk of HGD and the intestinal type of IPMN than patients with non-AP IPMN. AP effectively predicted highly differentiated heterogeneity with satisfactory sensitivity, specificity, positive and negative predictive values, and high accuracy.

A review of the included studies showed that the risk of malignancy in IPMN patients with a history of pancreatitis was significantly higher than in those without a history of pancreatitis [17–19, 25]. However, Roch et al. [26] did not find associations between AP history and malignancy in IPMN patients. Differences between populations in the included studies may have contributed to the inconsistent findings.

The results of the meta-analysis in the present study showed that AP is associated with highly heterogeneous IPMN and intestinal IPMN. The mechanism of AP development in IPMN is generally considered to be mucus production that leads to mechanical obstruction of the pancreatic duct, causing increased intra-pancreatic ductal pressure, rupture of the pancreatic vesicles, release, and activation of pancreatic enzymes, digestion of the pancreatic self-tissue, and activation of inflammatory factors that ultimately result in pancreatitis [27]. The described mechanism and the previous and present findings suggest that pancreatitis is associated with malignant IPMN. However, further study is still needed to identify associated factors between AP and malignancy in patients with IPMN.

The proportion of patients with a smoking history was significantly higher among non-AP IPMN patients than AP IPMN patients. Smoking is a known risk factor for pancreatitis [28]. However, Chavan et al. found a protective pancreatic effect of smoking against endoscopic retrograde cholangiopancreatography-induced pancreatitis, which may be due to the anti-inflammatory response of nicotine via the cholinergic anti-inflammatory pathway [29]. Recently, animal studies have also shown that nicotine reduces the severity of ERCP-induced pancreatitis by stimulating splenic T-cells, which play a protective role in the pancreas [30]. This mechanism, in particular, helps to explain the significantly lower proportion of smoking found in AP patients.

### Implications

The findings of this study highlight the importance of considering AP history when assessing the risk of malignancy in IPMN patients during clinical practice. Further research is needed to identify specific factors linking AP and malignancy in IPMN, which could potentially contribute to improved risk stratification and management strategies for patients.

### Limitations

The present study has several limitations, including the small sample size and data collected from only one center, which may include selection bias. The inherent restrictions of retrospective design also apply, including limiting the generalization of results to other populations and not allowing the measurement of certain factors or long-term follow-up. Protocol for the systematic review and meta-analysis was not pre-recorded on PROSPERO. Data on IPMN epithelial cell subtypes were missing for some patients. Lastly, the few studies in the meta-analysis may not provide sufficient power to draw reliable conclusions. The pooled analysis also included case-control studies with a lower evidence quality.

## Conclusion

The findings of our clinical data and the systematic review and meta-analysis suggest that patients with AP and IPMN are at a higher risk of presenting HGD and the intestinal type of IPMN compared to those without AP.

### Electronic supplementary material

Below is the link to the electronic supplementary material.

Supplementary Material 1

Supplementary Material 2

## Data Availability

All data analyzed during this study are included in this published article.

## Abbreviations

IPMN: Intraductal papillary mucinous neoplasm
AP: acute pancreatitis
ORs: odds ratios
CIs: confidence intervals
HGD: high-grade dysplasia
LGD: low-grade dysplasia
MGD: intermediate-grade dysplasia
HGD: high-grade dysplasia
IC: invasive carcinoma
