# Supplementary material for: Acute pancreatitis in intraductal papillary mucinous neoplasm: a single-center retrospective cohort study with systematic review and meta-analysis
Source: BMC Gastroenterol. 2023 Dec 1;23:424. doi: 10.1186/s12876-023-02972-4 (PMC10690977; doi:10.1186/s12876-023-02972-4)
Supplement: Supplementary file 2 — Supplementary Material 2 [file 12876_2023_2972_MOESM2_ESM.docx]

| **Supplementary Table 1. Postoperative follow-up of AP and non-AP groups in patients with IPMN.** | | | |
| --- | --- | --- | --- |
|  | **IPMN** | | **P-value** |
|  | **AP (n=11)** | **Non-AP (n=36)** |  |
| **Postoperative follow-up** |  |  |  |
| Length of hospital stay | 19.0 (15.0-27.0) | 28.0 (17.5-46.5) | 0.080 |
| Length of hospital stay after surgery | 8.0 (6.0-19.0) | 21.0 (11.0-39.0) | **0.014** |
| Complications |  |  |  |
| None | 10 (90.9) | 19 (52.8) | 0.184 |
| Fistulas | 1 (9.1) | 7 (19.4) |  |
| Bleeding | 0 (0) | 8 (22.2) |  |
| Infection | 0 (0) | 2 (5.6) |  |
| **Post-discharge follow-up** |  |  |  |
| Lost to follow up | 0 (0) | 1 (2.8) | 1.000 |
| Follow up period (months) | 18 (4.0-38.0) | 16 (3.0-33.0) | 0.718 |
| Outcomes |  |  |  |
| Tumor recurrence | 3 (27.3) | 1 (2.9) | **0.037** |
| AP recurrence | 0 (0) | 0 (0) | - |
| Pancreatic exocrine and endocrine insufficiency | 0 (0) | 5 (14.3) | 0.317 |
| Death | 2 (18.2) | 6 (17.1) | 1.000 |
| AP: acute pancreatitis; IPMN: intraductal papillary mucinous neoplasms. | | | |
| Continuous data are presented as median (Q1-Q3) and categorical data are presented as n (%). Significant values are shown in bold. | | | |

**Supplementary Table 2. Quality assessment of included studies based on the NOS**

| **Study** | **Selection (4)** | **Comparability (2)** | **Outcome (3)** | **Total NOS score** |
| --- | --- | --- | --- | --- |
| Tanaka 2020 | ★★★★ | ★★ | ★★ | 8 |
| Morales-Oyarvide 2015 | ★★★ | ★ | ★★ | 6 |
| Hata 2013 | ★★★★ | ★ | ★★ | 7 |
| Tsutsumi 2010 | ★★★ | ★★ | ★★ | 7 |
| Pelletier 2010 | ★★★ | ★ | ★★ | 6 |

NOS, Newcastle-Ottawa Scale (NOS).
